# The Interactive Work of Implementing Synchronous Video‐Conference Calls—A Qualitative Study Within Early Intervention for Infants With Childhood‐Onset Neurodisability

**DOI:** 10.1111/hex.70215

**Published:** 2025-03-19

**Authors:** Phillip Harniess, Anna Purna Basu, Deanna Gibbs, Jeff Bezemer

**Affiliations:** ^1^ Institute of Education, Faculty of Education and Society University College London London UK; ^2^ Great Ormond Street Hospital for Children NHS Foundation Trust London UK; ^3^ Faculty of Health and Life Sciences Northumbria University Newcastle UK; ^4^ Population Health Sciences Institute Newcastle University Newcastle UK; ^5^ Newcastle upon Tyne Hospitals NHS Foundation Trust Newcastle UK; ^6^ Barts Health NHS Trust London UK; ^7^ Queen Mary University London UK

**Keywords:** child disability, communication, early intervention, learning, multimodality, parents, service delivery, telehealth

## Abstract

**Introduction:**

This study explores the ‘peripandemic’ implementation of synchronous videoconference calls during COVID‐19 for delivering physiotherapy early intervention services to families of infants with childhood‐onset disability. The interactional experience of conducting early intervention through videoconference calls is under researched. We aimed to understand parents' and therapists' experiences of communication and learning within early intervention sessions for infants with cerebral palsy conducted via video conference calls.

**Methods:**

Data were collected through interviews, video case studies and focus groups involving 15 parents and 16 therapists. We used qualitative analytical methods inspired by grounded theory and multimodality.

**Results:**

Undertaking early intervention sessions via synchronous videoconference calls creates complexities and disrupts communication norms between parent, therapist and infant. These audio‐visual constraints have implications for developing shared understanding and learning. Resolving these challenges necessitated increased interactive work within the parent–therapist partnership. The onus placed on parents to have additional logistical roles in some circumstances created strain, which diverted attention from optimal learning.

**Conclusion:**

The post‐pandemic healthcare landscape pushes for digital innovation challenging traditional therapy models. Our contribution outlines that while videoconference calls may improve efficiency, they also add cognitive load and interaction challenges, which require modification to routine in‐person session designs. We provide recommendations for adaptive implementation strategies for videoconference calls that will benefit from further iterative codesign cycles.

**Public and Patient Contribution:**

We partnered with parents through public and patient involvement. Parents (*n* = 9) who were previous NHS early intervention service users formed the Parent Advisory Group (PAG). These parent partners came from a variety of backgrounds and provided their unique perspectives to directly contribute and guide decision‐making throughout the project. Their contribution influenced approach to recruitment and consent; the participant information and consent form development; topic guide development; considerations of the use of video in the project design and sense checking of analytical interpretations.

## 1 Introduction

Videoconference calls supported the continuity of access to physiotherapy early intervention during the COVID‐19 pandemic for families of infants with, or at risk of, childhood‐onset neurodisability. The experience gained through this accelerated adoption of videoconference calls during the COVID‐19 pandemic is being hailed as an (unintended) opportunity for innovating the delivery of child disability services [1]. This opportunity is connected with a desire to avoid defaulting to traditional models (post COVID‐19) as videoconference calls may engineer a positive disruptive catalyst to shift therapy provider and parent engagement expectations and behaviours towards empowerment practice models [2].

Telehealth encompasses the broader use of digital communication technologies to deliver healthcare services and support to families remotely, which may include low‐high tech strategies, involving asynchronous (e.g. sharing prerecorded video) and synchronous (e.g. real‐time videoconference calls) [1, 3, 4]. The movement to hybridisation (in‐person and remote telehealth) is also gaining research attention in child disability service delivery [5]. While different functionalities exist even within videoconference calls, within this paper we will focus on and refer only to synchronous videoconference calls [6]. Evidently, the pandemic did not allow forethought about optimising the experience of videoconference calls for both family and therapy providers. While research is growing into telehealth service design feasibility and acceptability, far less research is exploring what early intervention employing videoconference calls actually entails, how therapist and parents go about it and what challenges and opportunities arise in communication within this online platform.

We define communication as a two‐way interpretative act, which, importantly within a healthcare practitioner‐client environment, is inseparable from engagement and learning [7]. Communication in healthcare encounters across videoconference calls has different properties compared to in‐person communication [8, 9] but has an advantage over telephone consultations with regard to the ability to pick up visual communication cues [10]. Different healthcare videoconference consultations depend on physical examination to varying degrees and strategise by directing and cooperating with patients and/or family members to observe intended actions [11]. For example, in adult neurological and musculoskeletal physiotherapy settings the patient is more physically involved in establishing shared visible intelligibility of enaction, requiring collaborative communication strategies [12, 13]. Early intervention in childhood onset disability is likely to have some similarities and differences to these examples by virtue of its embodied enactive nature involving both the parent and infant in the home environment. Applied across diverse clinical settings, multimodality provides an appropriate theoretical lens through which to recognise and interpret the multifaceted nature of communication across different modes [7, 14, 15, 16, 17].

Snoswell et al. [18] assert that telehealth can be as effective as or more effective than usual clinical care but that efficacy is highly discipline specific. More research is called for to explore the collaborative opportunities of videoconference calls within different patient groups [19]. There is a little in‐depth real‐world exploration of the parent and therapist engagement experience in early years paediatric rehabilitation via videoconference calls [20, 21]. Therefore, we aimed to understand parents' and therapists' experiences of communication and learning within early intervention physiotherapy sessions for infants with cerebral palsy (CP) conducted via video conference calls.

## 2 Methods

This study is part of the larger Optimise EI project, which explores parent and therapist engagement in early intervention for infants with CP [22]. Using grounded theory methods, we examined key phenomena of interest. Given that the project took place during the COVID‐19 pandemic, this study specifically explored parent and therapist perspectives on communication and learning through videoconference calls in early physiotherapy.

### 2.1 Study Setting and Context

The study was UK‐based within the National Health Service (NHS). Three urban sites were chosen that provided community early intervention services within home and clinical settings. The sessions observed involved the organisation of tailored therapeutic activities incorporating play and using hands‐on support or equipment, to encourage the child's motor learning. In the immediate aftermath of the first lockdown in England, March 2020, videoconference calls were rapidly adopted to support continued early intervention service delivery. The sites used Microsoft Teams videoconference software. Once established, videoconference call sessions followed a similar structure as in‐person contacts. During video sessions, therapeutic actions were performed solely by the parent in their home, under the observation and guidance of the therapist.

### 2.2 Design

We applied grounded theory within a paradigm of pragmatism [23, 24, 25], which assumes that knowledge is historically and culturally influenced, therefore changeable and provisional. In alignment with our study, participatory approaches are supported by pragmatism, as the intersubjectivities of multiple stakeholders are incorporated in knowledge development to create implementable outputs for practice [26, 27]. Pragmatism also promotes flexible methods to address research aims [27]. For example, our interaction analysis was informed by multimodality, to support our identification of how therapists, parents and infants used different communicative modes to convey meaning in sessions [15].

### 2.3 Participants

Participants incorporated parents and physiotherapists and occupational therapists. Parents were included if their child was aged under 2 years corrected age, were at risk of CP or already diagnosed and were receiving early intervention services.

Therapists were required to have a minimum of 3 years post‐graduate experience in paediatrics. We used an initial purposive sampling frame for these specific identities and lived experiences of the phenomenon of interest within this study, and to scope diverse characteristics [28]. The initial sampling frame sought to select parent participants purposively to provide maximal variation in their characteristics. These characteristics included parent sex; age (infant and parent); family history; ethnicity; socioeconomic and educational status and infant medical history. With concurrent analysis, further sampling focused on identities that might provide alternative perspectives [25, 29]. For example, we purposively recruited fathers in later sampling, who orientated towards the practical aspects of videoconference calls. All eligible participants were approached by local site collaborators (therapists) directly and were provided with information sheets before informed consent was agreed and signed.

### 2.4 Procedure

We used mixed qualitative data collection across three overlapping phases. Data collection for the overall project covered a period from July 2019–February 2022. In phase one (July 2019–June 2021), 1‐1 and 1‐2 audio‐recorded online interviews, lasting 1–2 h, were conducted with parent(s) using a semi‐structured topic guide. During phase two (September 2020–January 2022), video case studies (involving parent, therapist and child) were undertaken. Within phase three (January–February 2022), two online therapist focus groups were conducted, lasting 90 min. Consistent with grounded theory, topic guides evolved with the progress of the study and to probe lines of inquiry [25, 30]. Examples of questions are provided from 1‐1 interviews and focus groups (supplementary document 1).

The phase two case studies included pairings between therapists, parents and their infant receiving intervention. The phase one parent interviews and phase three focus groups, while within the same services, were not specifically paired. Case studies support in‐depth exploration to address ‘how’ and ‘why’ questions in real‐life contexts [31, 32]. There were two data collection‐analysis procedures in the case studies, (i) video observation of early intervention physiotherapy sessions and (ii) participant follow‐up interviews using video extracts for reflective elicitation. As a result of COVID‐19 disruption to conventional in‐person intervention, the data from case studies for this paper included therapy sessions that were conducted via Microsoft Teams, lasting approximately 60 min. The criteria of data relevancy for analysis was decided on how it related to the phenomenon of interest—the exploration of engagement, communication and learning experiences of parent and therapist within videoconference calls. Follow‐up interviews, conducted separately with the parent and therapist, incorporated deeper reflections on the video footage. Video segments that were deemed to illustrate these experiences within the session were pre‐identified for discussion during interviews by participants and the researcher. An open elicitation ‘call out’ method was used, meaning during interviews participants could pause selected video segments to provide their real‐time reflection on observed events [16]. This participatory approach enabled stakeholders to highlight aspects of in‐session engagement meaningful to them.

### 2.5 Analysis

Interview audios were transcribed verbatim by a third‐party company. Data analysis was managed through NVIVO 12. Interview data were analysed following an interpretive descriptive approach and grounded theory principles, beginning with primary researcher immersion in the data, followed by open coding progressing to focused coding before making decisions relating to organising concepts [25, 33]. The video data were analysed similarly using inductive qualitative principles from a multimodal communication perspective [15]. Our observational analysis focused on the unfolding moments of interactions, recognising the relative resources afforded by the varied communicative modes through which actors shared meaning, such as through gesture; facial expression; posture; spatial positioning; embodied enaction and talk [15]. Immersion involved repeated watching of the videos, writing reflective memos and observational summaries before coding development. Constant comparison was used throughout, to contrast analytical development within and between participant accounts [29, 33] and video data analysis was triangulated with interview data analysis [34].

The presentation of quotes is pseudonymised within the results. For presentation of key video segments, transcriptions of the multimodal aspects of interaction were created using Psathas's convention [35], with the application of symbols deemed essential for data interpretation (File S2). The results involve the presentation of video vignettes as representative examples of the context integrated with interview data, to support conceptualisation.

We sought to establish trustworthiness in the design. The primary researcher analysed interview and video data and, independently, one researcher (JB) analysed the video data, and another researcher (DG) analysed four interviews [34]. Disagreements were discussed, and a consensus was found for early coding framework development, with discrepancies mostly related to semantics around coding development. Most discussions focused on comparing data to ensure a balanced presentation of organising themes. Wider team discussion supported more precise analytical decisions. The PAG also sense‐checked the presented data analysis. The primary researcher's reflexivity was enhanced through a reflective journal, to include consideration of positionality within interviews and its influence on analytical development. Memo writing enhanced analytical development.

### 2.6 Ethical Approval

This study received full ethical approval granted by the Bloomsbury NHS Research Ethics Committee (REC no. 19/LO/0298).

## 3 Results

Data are taken from 21 interviews with 15 parents (P) (from 11 families) and 3 physiotherapists (PT), including two case studies (CS) (using interview data and three videos of conference call sessions—approximately 180 min). Further focus group (FG) data involved 13 physiotherapists and occupational therapists. Table 1 provides participant characteristic information.

**Table 1:** Participant characteristics.

Parent Information		N	%
Parent carer mean age – years (range)		34.1 (18–50)	
Parent sex	Female	10	66.7
	Male	5	33.3
Parent ethnicity	Asian, British Asian	5	33.3
	Black African/Caribbean, Black British	2	13.3
	White British, White Other	6	40.0
	Mixed Ethnicity	2	13.3
Parent education	Degree or above	11	73.3
	A‐Level or equivalent (finished education 18‐years old)	1	6.7
	GCSE or equivalent (finished education at 16‐years old)	3	20.0
Child's presentation	At‐risk of cerebral palsy	2	
	Unilateral cerebral palsy	3	
	Bilateral cerebral palsy	6	
Mean child corrected age at initial interview – months (range)		11.9 (4–21)	
Household income (£)	< 20,000	2	20.0
	20,001–40,000	3	20.0
	40,001–60,000	1	16.7
	60,001–80,000	1	23.3
	> 80,000	2	16.7
	Not stated	2	
**Therapist information**			
Physiotherapist		11	73.3
Occupational therapist		4	26.7
Early years experience—mean years (range)		14.3 (3–31)	
Total post‐graduate experience—mean years (range)		17.9 (4–42)	
	Neurodevelopmental techniques (NDT)	9	
	Coaching	5	
	Sensory integration	3	
	Other neonatal training	3	
Relevant post‐graduate training	Other	2	

We present the data analysis in three interconnected codes: ‘Disrupted modes of communication’; ‘Collaborative communicative efforts for shared understanding’; and ‘The enhanced parental role’. Three case examples of short interactions are integrated to illustrate and support the analytical development.

### 3.1 Disrupted Modes of Communication

Parents and therapists highlighted the complexity of communication across videoconference calls. The video remove specific non‐verbal communication modes therapists are ordinarily skilled in using, including ‘bodily cues’ (embodied communication) and ‘where I might position myself’ (spatial orientation), while ‘maybe not using too many words, but just … demonstrate with my body what I want happening’ (CS1‐PT). Participants articulated how in face‐to‐face settings embodied ‘hand over hand’ guidance provided more ‘black and white’ understanding, compared to the communicative complexities created by conducting early intervention sessions via videoconference calls.

…in a conference call, you're not really supposed to interpret non‐verbal information … In a therapy session it's unique because you have to visually interpret as well as listen to an instruction and you have to physically interpret and help your child physically interpret what's happening on screen, right? And that is unique.

(Father, CS1‐P)

#### 3.1.1 Case Example One—Communicative Challenges

**Figure 1:**
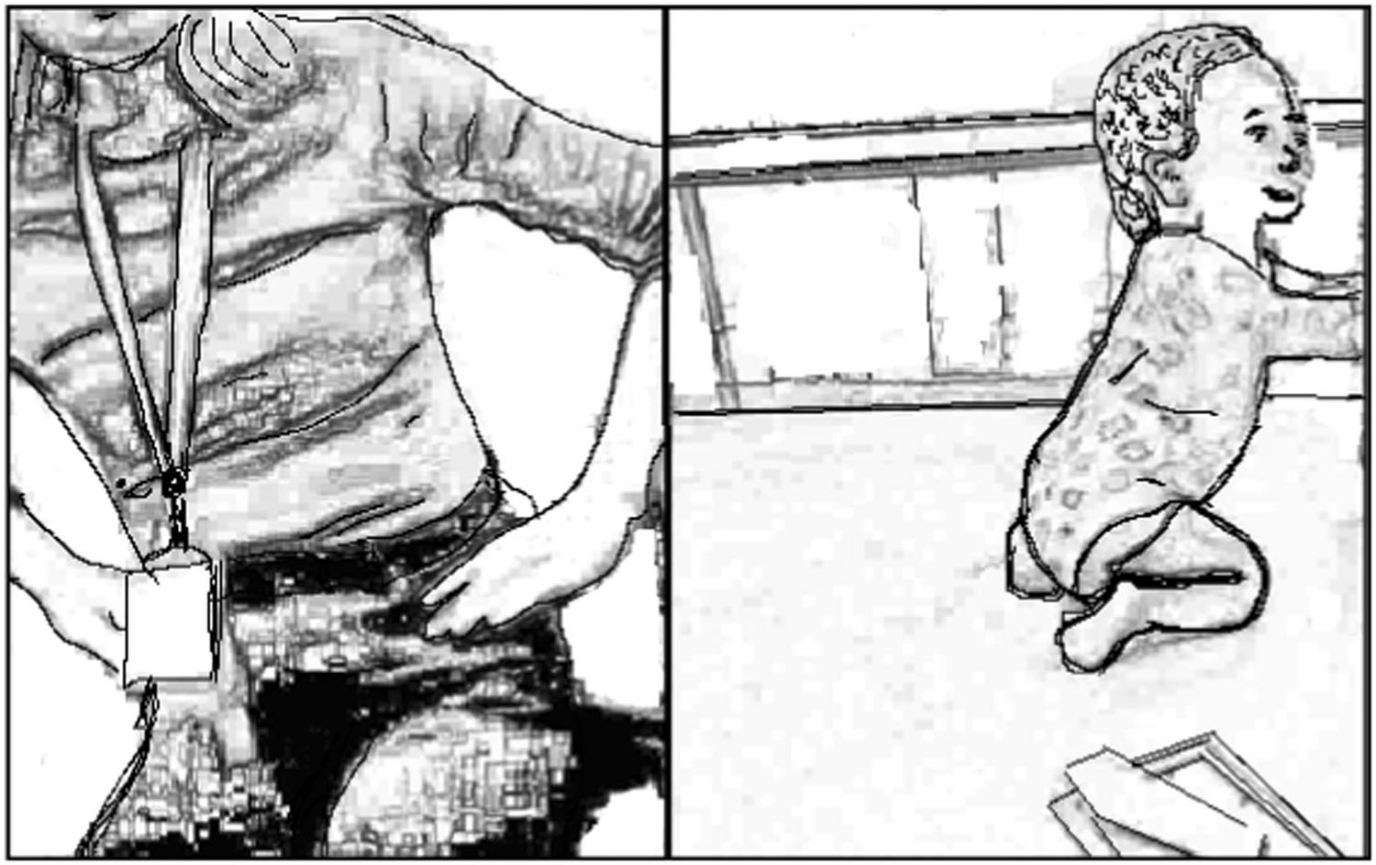
Therapist demonstrating task for the mother.

This video case example is representative of a difficult moment relating to establishing a shared understanding of a therapeutic task. It occurred over approximately 3 min, with the therapist focusing on encouraging the mother to try to support her child into a side‐sitting position. However, the mother appears hesitant to try this in case it upsets or hurts her child. After some time thinking (with pauses and cut‐offs in the talk), the therapist describes and demonstrates an alternative way to attempt the task, “… if your hands are on her hips, if you just bring her down sideways” (Figure 1).

The mother attempts the task, but the child crawls away to interact with the phone/camera and when the mother tries a second time, the child becomes upset. The therapist suggests one more attempt but the mother states.

I think that I don't know how to do it because I prefer you to show me *properly* because I don't know if I'm doing it right.

(Mother, CS2‐P)

The mother's tone indicated her frustration. In the follow‐up interview, the mother acknowledged that she was uncertain and underconfident in what she was being asked to do and feared causing her child discomfort—adding that she struggled to balance managing what the therapist was requesting with managing her own child's interests and strong agency. The therapist reassured the mother in the session, but the task was abandoned. The mother's final statement, ‘I prefer you to show me properly,’ revealed that their efforts to reach a shared understanding for joint action were insufficient. The mother corroborated in interview that her use of the semantic ‘properly’ was inferring her preference to be shown in‐person in a future session to remedy her understanding. On the same incident, the therapist reflected.

I think I … tried to explain something that was a bit new and that was clearly something that Mum felt more comfortable with me showing in person. At least she told me.

(CS2‐PT)

This example illustrates inherent challenges of communication and learning in early intervention where the therapist physical presence is removed. Namely these challenges pertain to embodied modes of communication and establishing and maintaining consistent shared audiovisual attention on each other's and the child's actions, while the parent manages the camera and child's behaviour. Part of the mother's hesitancy related to intervention in light of her child's muscle stiffness and the risk of causing her discomfort. She was uncertain about the haptic interpretation of how far she could ‘manipulate’ her child's position, without the demonstration by the therapist for a clearer understanding.

Other parents highlighted similar challenges with interpretation and translation of visual information into one's own orientation and embodied enaction with their child when following the therapist's removed visual guide via video.

… the difference I've found between the online, where you're just having an instruction, and even though the physio was demonstrating to us with the baby [dolly], because you're 2D, it was just impossible for us to understand it.

(Mother, P12)

Further visuoperceptual constraints were exemplified that impaired interpretation. For example, the image reversal feature in videoconference call software, which created confusion in the advice for treating an infant with unilateral CP.

… obviously, the phone is reversed … it's mirrored. So, we had the issue, I was doing it all on the wrong side and then I was like, ‘I think I'm strengthening his wrong side,’ and then I rang back and I was like, ‘… I think I've done it all wrong’ … this is the very beginning of them using … Zoom … and so we were learning together …

(Mother, P10)

Learning for both therapist and parent requires ‘accurate’ interpretation. Interestingly, these misunderstandings from communicative challenges sometimes created opportunities for collaborative problem‐solving, although more successfully in pre‐existing relationships, participants reasoned. Successful executions of therapeutic tasks were possible with persistence and ‘repair’ to communicative misunderstandings as the following example outlines.

##### 3.1.1.1 Collaborative Communicative Efforts for Shared Understanding

The complexities of communication via videoconference calls created a need for parents and therapists to manage their interaction in new ways to enable a therapy session to run as effectively as intended.

#### 3.1.2 Case Example Two—Collaborative Remediation

This video example presents an alternative scenario with the parent delivering a hands‐on therapeutic activity with the physiotherapist providing direct instruction. The mother begins by propping her mobile phone against the wall so that the therapist can observe, and the therapist verbally explains the task of supporting the child to pull to stand at the sofa through half kneeling. Initially, the mother attempts the task, but the child's quick movement from the floor into standing does not allow the mother time to intervene to support a half‐kneel transition as intended. The therapist seeing the difficulty with the mother's interpretation tries to remedy her understanding.

Physiotherapist: So Camila [pseudonym] if you can put‐ yeah. Can I show you what I mean, is that ok?

Mother: Yeah

Physiotherapist: Yeah. Ok Let me just‐ OK are you able to see my screen?

Mother: Yeah

Table 2 picks up the subsequent interaction. The therapist checks again that the mother can see the video on her mobile phone. The therapist embodies a half‐kneeling position for demonstration, including further verbal explanation of the task and the parent follows the instructions in real‐time from the therapist.

**Table 2:** Case example two—Therapist demonstrating activity.

Duration: 00:14:04‐14:34 (30 s) Line no.	
1. **T:** Alright can you still see me? 2. **M:** Yeah 3. **T**: Yep [OK when Emily's he:re:: if you could bring one leg forwards so: then 4. [((Demonstrates half kneel to standing action))] 5. she pushes up that wa:y (.) up to sta:nding. 6. But you‐ but you have to move your hands quickly before (.) she gets herself up (1.8) 7. **M**: ((Places toys on sofa)) 8. **T:** So: that's it perfect (.) so Emily's‐ (0.2) So when she's he:re‐ yeah‐ if you ho:ld‐ 9. **M:** ((Puts hand on child's right leg)) 10. **T**: Yep‐ Get one leg forward pe:rfect perfect! keep going‐ that's it (2.2) 11. [goo:d! Good! 12. [((claps and smiles))	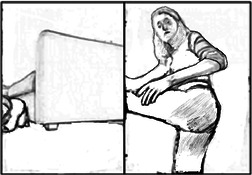 , 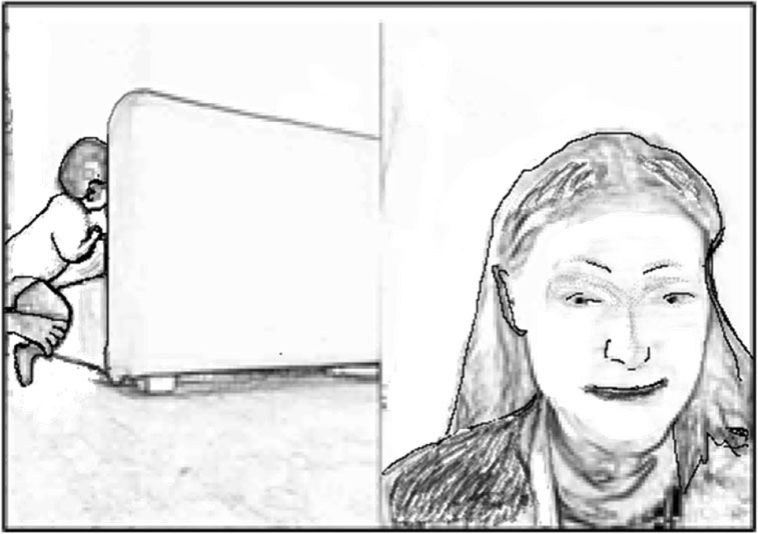

This example provides insight into the fundamental reality of a parent undertaking therapeutic tasks under the therapist's direction on videoconference calls and the challenge this poses. The therapist twice verifies that the mother is sharing her focus and can observe her on the screen, effectively ensuring a clear communication channel. The therapist needs to confirm because: (i) she cannot see the mother within the video frame to judge where her gaze is directed and (ii) the mobile phone placement across the room makes it more difficult for the mother to see the screen. Two attempts are taken to explain the task, first verbally and then including a physical demonstration, to remedy the mother's interpretation and reinforce the instruction, highlighting online‐specific communicative work for successful task execution to overcome videoconference call constraints. Hence, some communication modes necessarily become amplified, for example vocal tone and gesture.

Evidently, therapeutic activities via videoconference calls can be effectively completed. However, parents often described how the development of flow and momentum in sessions was disrupted by the logistics of coordinating the videoconference call while interacting with the child and therapist, including occasional technological interruptions, adding to communication and learning frustrations.

…in these video calls sometimes you're holding the camera but you're … chasing him to bring him back and you're losing that bit of momentum.

(Father, P4)

For therapists, the challenge was finding the optimum point when to intervene with guidance, as a verbal prompt might ‘break up the momentum’ as the child becomes distracted by the therapist's voice, meaning therapists ‘have to pick [their] moments [to speak]’ (CS1‐PT).

#### 3.1.3 Case Example Three—Parent Leading to Enable Session Momentum and Flow

Therefore, to sustain momentum one father often took more initiative to provide direction within sessions rather than wait for the therapist. The following example presented in Table 3, is representative of the father's initiative as he leads in setting up the activity for the therapist to observe and provides commentary of the activity (commonplace for this parent‐therapist partnership). The father directs his conversation toward the child as if to explain to him at length what he would like him to do. He clarified in interview that this provided a narrative of events to support the therapist's observational interpretation (rather than for the child's benefit) as he was aware of the limitations of what the therapist could see. The therapist adopts a supporting role, by interacting with the child to maintain his interest using voice and gesture. The father's final comment ‘look at that nice grip…I like that’ provides his own evaluative summation of a successful performance of the child, also suggesting where the father wants to draw the therapist's attention to.

**Table 3:** Case example three—Father displaying child behaviours and providing commentary for therapist.

Duration: 00:31:48 − 00:31:53 (25 s) Line no.	
1. **T**: I can't quite see the right the:re (.) Warren I don't know if= 2. **F: =**Alr[ight ((adjusts laptop)) 3. **T:** [Ah thank you very much 4. **F:** So shall we do it like this instead? Shall we start the book again 5. And what I'd like you to do is you can point a::ll you like with that hand 6. but we're going to ho:ld the book with our right like thi:s. ((supports child's right hand to hold book)) 7. That's it (.) a nice resting position 8. Can you point to the house? Or the [shapes 9. [((child turns multiple pages)) 10. or just run all the way through the book. 11. O:h loo:k at the bu:tte:rfly!= 12. **T**: =[O:h loo:k at the bu:tte:rfly:! 13. [((gestures butterfly sign)) 14. **F:** Look at that [see (.) 15. [((points to child's hand)) 16. Look at that nice little grip we've [got there 17. **T:** [Bu:tte:rfly:: 18. **F:** That's something? I like that.	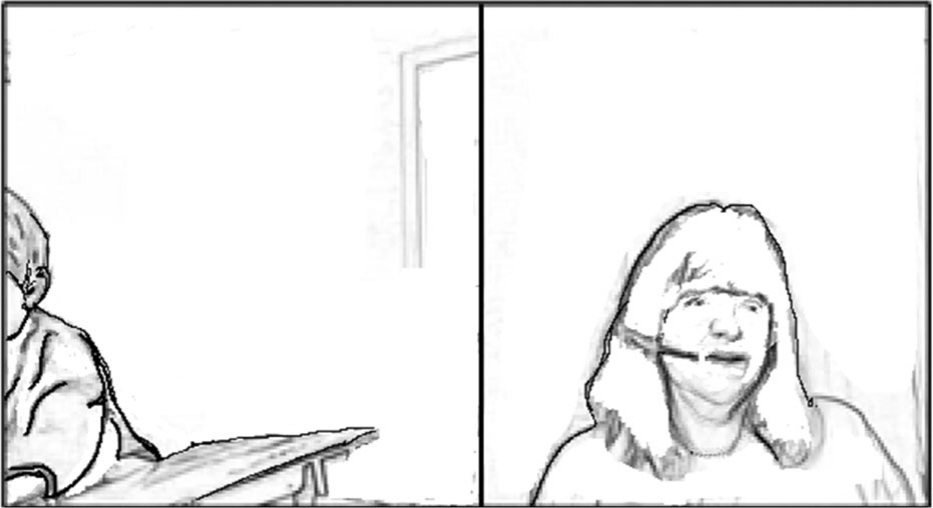 , 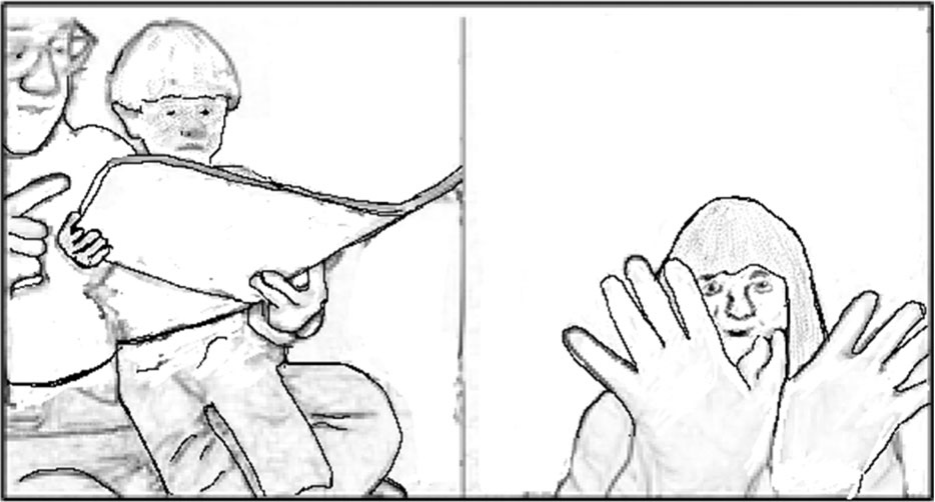

##### 3.1.3.1 Staging and Voice‐Over

As the examples illustrate, different parents may take on contrasting approaches within video conference calls. The mother continued to look for greater therapist direction until it was incompatible, and the father took a lead to enable the session to flow effectively. In the circumstances of undertaking videoconference calls, some parents indicated that they provided a ‘more performative version of what you might do in the room’ (CS1‐P) when being observed by the therapist on videoconference calls. This contrast was deemed necessary to compensate for the therapists' limited ability to visually interpret events, position themselves in the environmental space, be guided by the child's movement and support at the right moments. With embodied assessment and treatment removed for therapists, frustration occurred for some parents (e.g. case example one). For others, parents provided ongoing commentary (e.g. case example three), describing and explaining movement, including the haptic (‘feel’) of movement in collaboration with the therapist. It was observed that parents giving the ‘session a bit of a voice over’ offered more subtle communication within the flow of sessional. For example, to acknowledge (verbal) and then integrate therapist's guidance with forward planning at appropriate moments to avoid disrupting the child's engagement. Alternatively, where less parental commentary was provided, the therapist needed to make more communicative efforts to minimise misunderstandings (example two). Also, therapists expressed their occasional concern about safety with the absence of their own embodied interpretation.

… we have to just say how did it feel? Because we couldn't feel the tone or whether you think baby was safe … I hated it.

(FG‐PT2)

Parents expressed that ‘it's like being on a stage’ when demonstrating therapeutic activities ‘to the audience [therapist],’ which was augmented by the projection of their own image back to them. Some parents explained that they mirrored the intentions and energy of the therapist within the room. They staged play activities of assigned therapeutic activities to engage the child and to provide demonstration for the observing therapist to elicit feedback, validation or new ideas from the therapist (such as case example three). However, the performance was fragile in many ways, particularly when the child did not perform for the camera. Overall, for more successful collaborative engagement, parents were required to lead activities and work harder in their interactive play to maintain the child's engagement by proxy of the therapist.

##### 3.1.3.2 Camera Work

**Figure 2:**
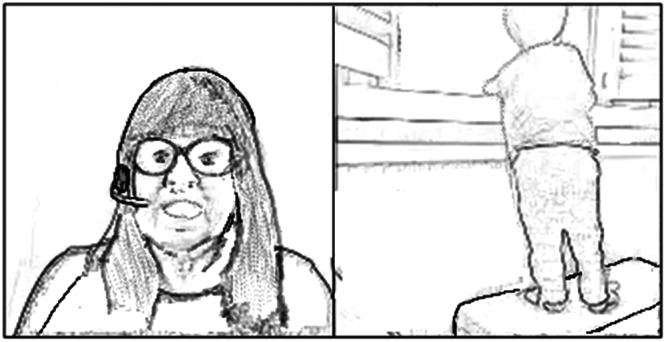
Example of frame boundary creating safety concern.

The parents' role demanded camera direction. Decisions about the positioning and use of the camera could, to a greater or lesser extent, affect therapists' meaning making from what they observed and heard. When ‘your camera doesn't have any agency,’ parents needed to orchestrate (parts of) the session by deciding what was in the frame, the angle of the shot and controlling the interface of the platform while attending to and giving directions to the child. Preparation was required for the technological‐environmental space for the camera shot by having props available for integration within the session and to support the session's flow. Parents described preparation that included splitting up the space/room(s) and having toys on hand, considering the furniture that will be required for activity, or to place their device upon, and including deliberation of the distance between prospective activities and the camera to optimise the view. Examples two and three involved requests by the therapist for the camera angle to be adjusted, indicating the frame limitations. In example two, the physiotherapist had difficulty seeing the mother's actions, when she was out of frame, which affected her perception of how responsive the mother was to the child's actions. In the therapists' focus groups, it was discussed how the boundaries of the camera lens constrained a broader appreciation of the home environment, space or equipment available to the child or parent for use in the session. In interviews, parents and therapists emphasised how the inability of therapists to observe the home environment outside of the frame meant that opportunities were missed to incorporate aspects of this into treatment or advise around equipment and, in some circumstances safeguarding considerations. In a separate video example, the physiotherapist had concerns (due to the camera framing) as to the closeness of the father to offer safe handling to the child in a potentially risky activity with the child standing on the bench (Figure 2), incidentally, the father was next to the child just outside of shot.

In other examples, the mobile child needed to be followed as they moved out of frame.

… finding a place for the phone, so she [physio] can see everything. He's crawling around, I'm trying to talk to her, help him, I just feel like it's [in‐person] a bit safer because, if he's doing the exercises, and then I'm also trying to talk to her, or look at the phone, or make sure that she can see what he's doing … [it] makes life … more difficult, you're multi‐tasking more.

(mother, P10)

The child's movement created a constant requirement to anticipate and adjust the camera to incorporate the child's movement to enable the therapist to continue to interpret what was occurring. These logistical processes, with the parents trying to direct the session using the camera, placed increased demands on them to concurrently consider the therapist's visual perspective through the camera placement. Hence, parents often felt that having two people present was preferable, one doing the camera work and the other performing therapy, to enable effective engagement and learning but this was not possible for more isolated parents.

During therapeutic activities involving the child, parents often chose low angles so that the therapist could feel more involved and observe what was occurring. This angle had the added benefit of enabling the therapist to support the child to engage in more relational interaction through the screen. Although, the close‐up shot presented another challenge, where the child easily became distracted by the opportunity to interact with the technological device (example one). The device had to be placed far enough away to prevent this, but still close enough to create a sense of relational engagement. In other situations of reciprocal feedback (not involving therapeutic activity) an eye‐level angle was adopted. Audio was also a consideration. Participants highlighted how conversational turn‐taking was tested by latency on videoconference calls, where ‘conversations start to overlap quite quickly, and people don't hear each other.’ Hence, specific accommodations to enable ‘flow’ were required, for example, clearer pauses between speaking and exaggerating facial gestures when wanting to interject.

### 3.2 The Enhanced Parental Role

Unquestionably there was an increased reliance placed upon parents to take on an enhanced active role with the demands of logistical multi‐tasking to implement a ‘successful’ videoconference call session. Therapists and parents hypothesised that this shift to more active involvement could create increased opportunities for parental practice and learning within sessions, for carry‐over. Nevertheless, many parents found the increased role expectancy unsettling. Therapists were also concerned about the pressure these circumstances created.

I think there was a sense of onus that was put on the parent that wasn't negotiated. It [videoconference calls] was just forced on them and so … I was doing loads more reassurance, but it was along the lines of, ‘this is the best we can do’… It wasn't ‘this is ideal’.

(FG‐PT1)

Rather than supporting optimal parental engagement and learning, the role strain created by the multiple demands was reportedly distracting and detracted from potential learning opportunities. Even parents who appeared to cope more confidently with the enhanced role expressed difficulty undertaking reflective learning when also managing the session on videoconference calls. For some, engaging via videoconference calls became a stressful burden within sessions.

… each week we would dread the time. And [she] would often … be crying … we weren't able to enjoy the time … And then we'd have the technology that was always going in and out … we found it quite stressful.

(Mother, P6)

In contrast, some parents considered potential benefits of hybrid models incorporating videoconference calls. They valued the opportunity to reduce time spent travelling to appointments and the reduced disruption to their child's routines. One parent felt interim online sessions could be shorter (than in‐person) and used to provide focused shared observation with the therapist of their child's progress on task‐specific goals. This approach was also felt that it might provide more flexibility to follow the child's lead. However, many therapists had not considered this potential innovation and continued to attempt to directly translate routine in‐person sessional design to an online context. In these circumstances, therapists reported that frequently online sessions were deemed so inadequate that additional in‐person sessions were required. Therefore, many therapists reported abandoning videoconference calls following the pandemic with families and did not attempt to explore adaptations or co‐designed alternative hybrid approaches, as suggested by some parents.

## 4 Discussion

Our study aimed to understand parents' and therapists' experiences of early intervention mediated via video conference calls for infants with CP, centring on communication and learning. The findings highlight that communication and learning are disrupted during parent and therapist engagement via videoconference calls, which increases the need for collaborative efforts and potentially increased parental involvement.

A significant contribution of this paper is to illustrate how the audio‐visual characteristics of early intervention physiotherapy sessions conducted via videoconference calls create new challenges across multiple modes, including embodied enaction, haptics and spatial orientation. The video becomes the sole communication challenge as it mediates the audio and visual interaction while making these other resources, such as direct touch, unavailable. The audio creates a time‐lapse, and the video creates a frame and renders everything two‐dimensional. These communicative challenges, therefore, impact upon ‘success’ within the therapy sessions and create frequent misunderstandings with the need for collaborative work to ‘repair’ during interactions [36]. There are frequent negotiations and attempts to establish and maintain a shared visual focus. Meta‐conversations establish a ‘channel’ (e.g. “I can't see … can you move the camera”) [9, 37]. Participants regularly need to reorganise themselves and the camera, especially around the moving child. Conversational turn‐taking is a greater consideration with the therapist's timing of interventional talk to avoid interrupting the child‐parent interaction, when the parent's gaze is not towards the camera during those moments. The therapist's restricted access (with sight and touch) of the child and parent, hampers their ability to observe and touch to assess the child's and subsequently intervene in an embodied way, occasionally creating some safety concerns, a finding also observed by others [12].

While both parent and therapist need to increase their interaction work, parents face a greater challenge to take on more enhanced roles and communicative responsibility within this partnership, to mitigate against specific disadvantages of the online context. Our data indicated that, within a videoconference setting, parents are hyperaware of the staging and exhibiting of desirable and learned behaviours to the observing therapist, who themselves monitor and make sense of parent and child behaviours from a distance to provide guidance. It is feasible that in these circumstances parents may feel the need to prove their competence, which may, not only be stressful but also shape their interactions and openness with therapists [38, 39, 40].

Another example of the enhanced parent role surrounds camera use, and, while camera positioning may be negotiated between actors, in real‐time parents are often making the decisions. Decisions parents make about the camera position become a communication mode, which may influence the therapist's interpretation of events (whether knowingly or unknowingly to parents). This phenomenon aligns with film and multimodality theory, which considers how visual intelligibility and the viewer's visuoperceptual interpretation are created by the decisions of the director [41, 42]. Perspective, or camera angle, is a well‐known semiotic practice that affects subjective viewer interpretation [43]. Parents often chose close‐up shots to bring the therapists closer to the action; within film theory, these angles are also used to demand a closer relationship of the viewer through a more profound emotional engagement than a distant shot [41]. Alternatively, eye‐level shots engender a perception of trust for the viewer e.g. within news reporting [44], and interestingly within the sessions, parents and therapists operated with this angle for reciprocal feedback.

### 4.1 Implications

The rationale of ‘not returning’ to normal following the pandemic centres on the premise that the disruption offered a unique opportunity to accelerate digital solutions to improve healthcare efficiency and challenge traditional professional‐parent relational positionality [2]. Arguably, the practice observed in our study assumed that routine in‐person sessions could be directly translated to a videoconference call context and insufficient reflection was given towards adaptation to a more implementable form of delivery for all concerned. For example, an adaptation might consider shorter sessions focused on the execution of one task in the home context with the child leading and the parent supporting, which also aligns with best practice guidelines [45]. The reason for the lack of imagination around adaptation in our study could be related to the embedded habits and routines associated with traditional expert‐physiotherapy models, which emphasise expert clinician handling [46, 47]. The effectiveness of these approaches is debated, not least for their undermining influence on parental self‐efficacy development [47, 48, 49, 50]. Therefore, the disruptive effect of removing handling, by videoconference calls, is considered by some as beneficial, particularly for promoting closer collaboration between therapist and parent [20, 51, 52]. With traditions yet to be established in online early intervention practice, it provides a platform for parents and therapists to reevaluate and codesign their roles and pedagogic approach [1, 2, 5, 52]. As adopters, therapists will require sustained identity (re)formation to adjust their professional cultural beliefs and make the necessary behavioural change for future embedding and adaptation of successful telehealth implementation [52, 53, 54, 55, 56]. To support this process we have summarised implementation considerations from our findings (Table 4).

**Table 4:** Implementation considerations based on findings.

Target area	Recommendations
Adapted sessional designs	Use a hybrid model, with videoconference calls implemented for review and progression of pre‐agreed home activities Consider more frequent sessions that are reduced in length and that have focus on a participation in a daily care tasks e.g. dressing or play moments in the home Recognise the opportunity for parents and child to practice greater autonomy within leading these moments, if they desire Where parents find it challenging to undertake practical therapeutic tasks via videoconference calls, reorientate the sessions towards broader supportive therapeutic conversations
Environment, ‘camera work’ and device	A laptop is preferable to a mobile phone to enable a more optimal framing of activities and the ability for parent and child to see and interact with the therapist The timing of the session may need to be arranged when the parent can receive the support of another person to direct the camera Consider camera positioning to support optimal visual interpretation and communication ‐ before the session work out with parents the environmental set‐up and device placement to support a view of the action in different positions Placement of the device low and close‐up within the ‘action’ will support therapist observation and interaction with the child (while also avoid getting too close, so the child is not distracted by the device) Use eye‐level perspective to support relational dialogue and feedback between the therapist and parent
Foundational relational context and meta‐conversation around codesign	Pre‐existing parent‐therapist relationships developed through face‐to‐face interactions are better placed to deal with the complexity of navigating communication within videoconference calls Create space for conversation about the role of the videoconference call in the therapy delivery, outlining its potential affordances and limitations Negotiate expectations and create initial co‐designed aims of the sessions with built in flexibility to adapt the approach to meet these aims or re‐evaluate the role of sessions within the therapeutic partnership Individualisation is required according to parental circumstances relating to: digital access and literacy; other siblings present; available social support and other potential pre‐existing stressors

Our findings have also problematised early intervention videoconference call delivery in how this medium adds communication complexity to sessional activities. Fundamentally, parents cannot be passive, with a greater onus placed upon parents to adopt more active roles, demanding greater interactive work and confidence. In an early intervention context, parents are processing a difficult parental transition, which can affect engagement readiness [57]. Not all parents feel fully able to meet the challenge of conducting online sessions for reasons of confidence, capability and capacity according to their available resources (e.g., social support, reliable technology and digital literacy). Materially, if parents only have access to a mobile phone, then their learning experience will be inferior to using a laptop [58]. Latency, exacerbated by bandwidth, has similarly been raised by others [9, 10]. These factors could explain why many parents and therapists found the additional complexity of videoconference call access stressful during this period, which concurs with other pandemic‐related telehealth experiences [55, 59]. The expectation placed on parents to take on more responsibility risks exposing inequities as some parents are likely better placed to optimise access with enhanced communicative and technological skills/experience [3, 60]. Therefore, design optimisation must avoid creating a two‐tiered telehealth provision caused by a digital access divide, which would compound family health inequities [3, 58, 61].

In addition to our recommendations (Table 4), frameworks have been developed to support therapists in implementing family‐centred approaches within telehealth more broadly, including coaching integration [6, 62]. Preparation of both therapist and parent, by openly discussing realistic expectations, roles and session design within videoconference call sessions will help reduce stressors, functional barriers and improve access experience, including parents from diverse backgrounds [62, 63, 64].

### 4.2 Limitations

The main limitation relates to the small amount of video data informing the findings, due to its exploratory nature and incidental collection across the pandemic period. However, as little research on interaction via videoconference calls in early intervention exists it is a beneficial starting point that implicates further research to build on our formative understandings.

Data was collected before, during and after the COVID‐19 pandemic. The focus group involved clinicians reflecting on their practice during this time, which may have included an element of recall bias. We were able to consider whether recall was problematic through triangulation of findings with our concurrent analysis within the broader data set, which included clinicians within the case studies. We concluded that themes drawn out in focus groups had strong support from other data analyses.

Opinions differ on whether video observation distorts interactions or behaviour [65]. While awareness of being observed may influence presentation, impression management is routine in clinical practice [66]. There is no reason to assume that with their knowledge of being videoed, participants would or could be able to perform at their ‘best’, assuming they (or we) know what best is and could choose accordingly [67].

## 5 Conclusion

Our study captured early aspects of a real‐time ‘peripandemic’ normalisation process of adoption‐implementation of videoconference calls in routine early intervention physiotherapy clinical practice for infants with childhood‐onset neurodisability [62, 68, 69]. Our contribution outlines that while videoconference calls may improve efficiency, they also add cognitive load and interaction challenges, which require modification to routine in‐person session designs. We provide recommendations for adaptive implementation strategies within videoconference calls that will benefit from further iterative codesign cycles.

## Author Contributions

**Phillip Harniess:** conceptualization, investigation, funding acquisition, methodology, visualization, data curation, writing – review and editing, writing – original draft, formal analysis, project administration. **Anna Purna Basu:** conceptualization, funding acquisition, writing – review and editing, supervision, methodology. **Deanna Gibbs:** conceptualization, funding acquisition, writing – review and editing, methodology, supervision, formal analysis. **Jeff Bezemer:** conceptualization, funding acquisition, writing – original draft, investigation, methodology, formal analysis, writing – review and editing, supervision, visualization.

## Conflicts of Interest

The authors declare no conflicts of interest.

## Supporting information

Supporting information.

Supporting information.

## Data Availability

The data that support the findings of this study are available on request from the corresponding author. The authors have nothing to report.
