# Supplementary material for: The Interactive Work of Implementing Synchronous Video‐Conference Calls—A Qualitative Study Within Early Intervention for Infants With Childhood‐Onset Neurodisability
Source: Health Expect. 2025 Mar 19;28(2):e70215. doi: 10.1111/hex.70215 (PMC11922807; doi:10.1111/hex.70215)
Supplement: Supplementary file 1 — Supporting information. [file HEX-28-e70215-s001.docx]

Supplementary Document 1

# Example of topic guide questions

Note: The interview schedule changed iteratively over the study, according to findings from the qualitative fieldwork. The questions and prompts were tailored to the specific answers of each interviewee. The interview schedule given here is therefore a general topic guide for the types of topics in the qualitative interviews.

## 1-1 Parent Interview:

| **Topic covered** | **Questions** |
| --- | --- |
| Scene setting  Parents’ early experience and understanding of their child’s condition | Can you tell me about your and your baby’s early experience and how that led to receiving early physiotherapy and OT? |
| Parent outcome expectancy and their role/involvement | How would you describe what optimal involvement/engagement looks like for you in EI?  What are your expectations of therapy (therapist engagement) for your baby?  What are your expectations for your role and involvement in the therapy? |
| The therapy session | What does a typical therapy session look like?  If sessions are on video calls, can you describe them?  What works well?  Is there anything that is challenging within sessions? |
| **Influence of shift to video calls** | Is there any difference in your role or are involved in sessions since adopting the videoconference calls? How?  In your opinion, does the video call influence communication? How?  Can you reflect on the benefits/drawbacks of this change? |

## Physiotherapy and Occupational Therapy Focus group:

| Topic covered | Questions |
| --- | --- |
| Scene setting  Beliefs/ attitudes regarding parental engagement | What do you think optimal parental engagement in EI looks like?  What do you think enables and constrains parents to be able to optimally engage in EI? |
| Expectations of role | What do you perceive are parents’ expectations, regarding how they want to engage / their role?  What are your strategies to support parental engagement in sessions?  How do you feel that your approach in sessions influences parental participation/engagement?  How is this affected by the setting e.g. at home in-person versus online videoconference calls? |
| **Influence of shift to video calls** | How did the shift to online sessions affect early intervention sessions?  **Prompts:**  How did the shift to online sessions affect your role?  How did the shift to online affect communication, if at all?  How do you think this shift affected parents and their baby?  What do you feel was the result of the shift? |
| EI context – transition and CP diagnosis | Do you feel that parental engagement might change/ transition over and during EI period? How?  How would that influence your approach at different points in that process? |
| Priming for recommendations | What key skills do you think would help therapists towards engaging with parents in EI, across the areas we have covered today? |
