# Supplementary material for: The Interactive Work of Implementing Synchronous Video‐Conference Calls—A Qualitative Study Within Early Intervention for Infants With Childhood‐Onset Neurodisability
Source: Health Expect. 2025 Mar 19;28(2):e70215. doi: 10.1111/hex.70215 (PMC11922807; doi:10.1111/hex.70215)
Supplement: Supplementary file 2 — Supporting information. [file HEX-28-e70215-s002.docx]

## Supplementary Document 2

# S2: Video transcription glossary

| **Symbol** | **Descriptor** |
| --- | --- |
|  | **Sequencing and timed intervals** |
| [  [ | Overlapping conversation (single left-hand bracket) |
| [ ]  [ ] | End of overlapping conversation (single right-hand bracket) |
| =  = | Latching – no interval between two person’s talk |
| (0.6) | Silence or pauses between conversation (time number in brackets) |
| (.) | Micro pause |
|  | **Characteristics of speech** |
| : | Sound stretch (colon after) and elongated (multiple colons) |
| - | Cut-off i.e. noticeable abrupt stop (dash) |
| . | A stopping fall in tone |
| , | Continuing intonation |
| ? | Rising intonation |
| ! | Animated tone |
| ˚ ˚ | Soft tone |
| ↑ ↓ | Marked rising and falling intonation |
|  | **Presentation convention** |
| (( )) | Researcher’s description of relevant feature |
| … | Partial omission of speakers’ utterance |
| 1.  2. | Line numbers |
